# Catalytic activities of noble metal atoms on WO_3_ (001): nitric oxide adsorption

**DOI:** 10.1186/s11671-014-0713-2

**Published:** 2015-02-11

**Authors:** Xiaoyan Ren, Shuai Zhang, Chong Li, Shunfang Li, Yu Jia, Jun-Hyung Cho

**Affiliations:** International Laboratory for Quantum Functional Materials of Henan, and School of Physics and Engineering, Zhengzhou University, Zhengzhou, 450001 China; Center for Clean Energy and Quantum Structures, Zhengzhou University, Zhengzhou, 45001 China; School of Mechanical and Electrical Engineering, Henan Institute of Science and Technology, Xinxiang, 453003 China; Department of Physics and Research Institute for Natural Sciences, Hanyang University, 17 Haengdang-Dong, Seongdong-Ku, Seoul, 133-791 Korea

**Keywords:** Surface, Catalytic, Charge transfer, Bond length

## Abstract

Using first-principles density functional theory calculations within the generalized gradient approximation, we investigate the adsorption of NO molecule on a clean WO_3_(001) surface as well as on the noble metal atom (Cu, Ag, and Au)-deposited WO_3_(001) surfaces. We find that on a clean WO_3_ (001) surface, the NO molecule binds to the W atom with an adsorption energy (*E*_ads_) of −0.48 eV. On the Cu- and Ag-deposited WO_3_(001) surface where such noble metal atoms prefer to adsorb on the hollow site, the NO molecule also binds to the W atom with *E*_ads_ = −1.69 and −1.41 eV, respectively. This relatively stronger bonding of NO to the W atom is found to be associated with the larger charge transfer of 0.43 *e* (Cu) and 0.33 *e* (Ag) from the surface to adsorbed NO. However, unlike the cases of Cu-WO_3_(001) and Ag-WO_3_(001), Au atoms prefer to adsorb on the top of W atom. On such an Au-WO_3_(001) complex, the NO molecule is found to form a bond to the Au atom with *E*_ads_ = −1.32 eV. Because of a large electronegativity of Au atom, the adsorbed NO molecule captures the less electrons (0.04 *e*) from the surface compared to the Cu and Ag catalysts. Our findings not only provide useful information about the NO adsorption on a clean WO_3_(001) surface as well as on the noble metal atoms deposited WO_3_(001) surfaces but also shed light on a higher sensitive WO_3_ sensor for NO detection employing noble metal catalysts.

## Background

NO*x* gases such as NO and NO_2_ which are produced from the reaction of nitrogen and oxygen gases in the air during combustion damage not only our environment including air pollution and land contamination but also human health. Therefore, it has attracted much attention in recent years to develop a high-performance NO*x*-sensing equipment [1-4]. For the detection of NO*x*, a number of gas sensors using semiconducting metal oxides such as ZnO [5-7], MoO_3_ [8,9], In_2_O_3_ [10], SnO_2_ [11,12], TiO_2_ [13], and WO_3_ [14] have been reported theoretically and experimentally.

Tungsten oxide (WO_3_) has many unusual properties which make it suitable for various applications, e.g., high sensitivity of reducing and oxidizing gases [14], excellent electron transport and photosensitivity, and high stability-resisting photocorrosion in aqueous solvent [15-20]. Especially, WO_3_ sensors have been widely applied for the detection of NO*x* gases [21-23]. In order to enhance the performance for NO_2_ detection, WO_3_ sensors have utilized the addition of metal atoms [24,25] as catalysts. However, there have been relatively few reports for the WO_3_ sensor detecting NO molecule [26,27], and furthermore, theoretical studies for the adsorption of NO on WO_3_ surfaces are still lacking. In this sense, an accurate first-principles density functional theory (DFT) calculation for the NO adsorption on WO_3_ surfaces is highly desirable for the application of WO_3_ sensors to NO detection.

**Figure 1:**
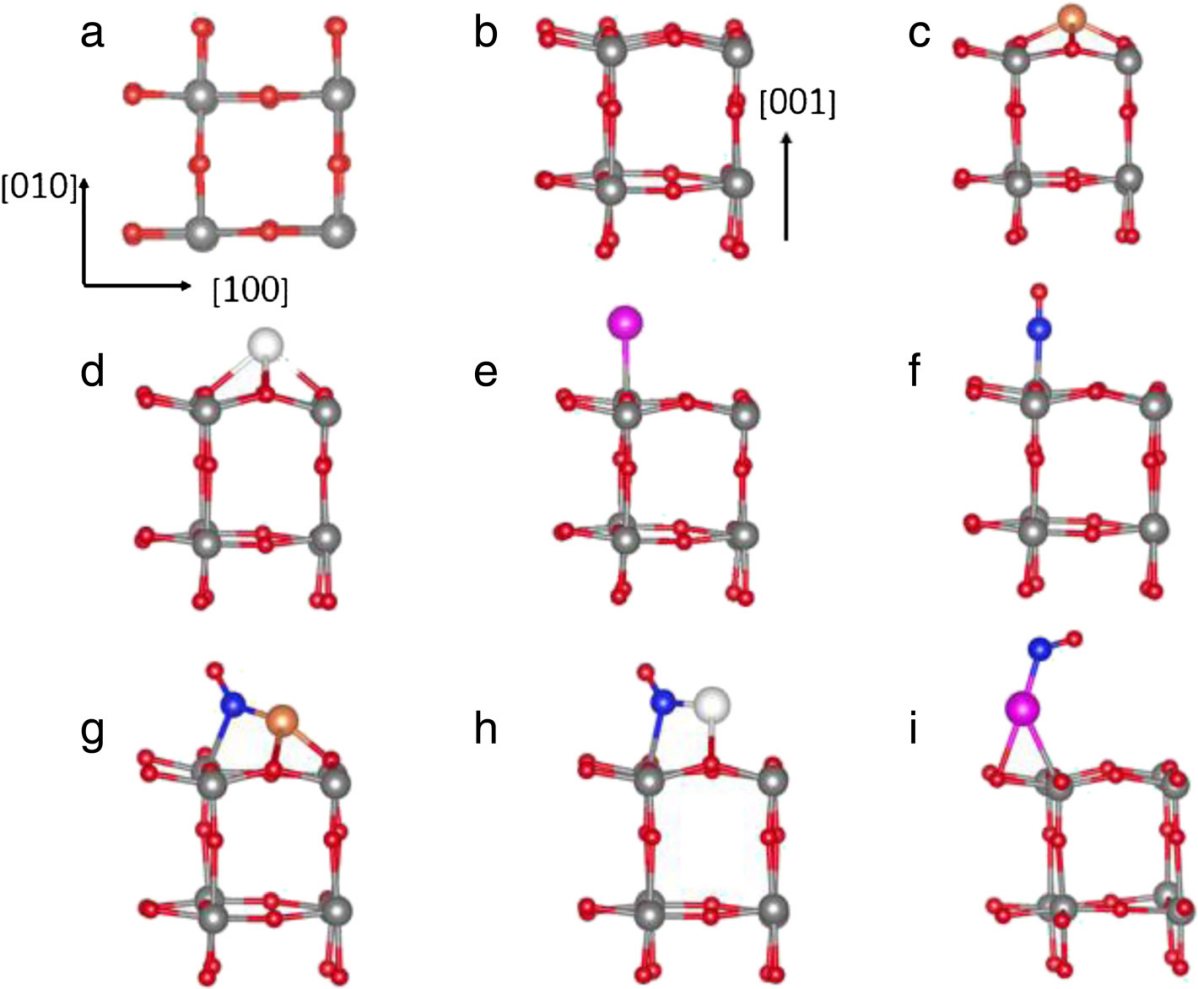
**Optimized atomic structure. (a)** Top view and **(b)** side view of the clean WO_3_ (001) surface. The large and small circles represent W and O atoms, respectively. The most stable structures for the Cu, Ag, and Au atoms deposited on WO_3_ (001) surface are displayed in **(c)**, **(d)**, and **(e)**, respectively. The most stable structures for NO adsorption on a clean WO_3_ (001) surface and the Cu-, Ag-, and Au-deposited WO_3_ (001) surfaces are displayed in **(f)**, **(g)**, **(h)**, and **(i)**, respectively. In **(f)**, **(g)**, **(h)**, and **(i)**, the circle for N atom is larger than that for the O atom.

In this work, we perform a first-principles DFT calculation to investigate the adsorption of NO molecule on a clean WO_3_(001) surface as well as on the noble metal atom (Cu, Ag, and Au) deposited WO_3_(001) surface. Here, the (001) surface (see Figure 1a,b) of γ-monoclinic WO_3_ is taken into account because it is the most stable at room temperature [28]. We demonstrate that the Cu-, Ag-, and Au-deposited WO_3_(001) surfaces exhibit different catalytic behaviors for NO adsorption, that is, the magnitude of adsorption energy (*E*_ads_) is in the order of Cu > Ag > Au. This different binding behavior of NO on WO_3_(001) depending on the noble metal species can be traced to the difference in charge transfer from the substrate to adsorbed NO molecule. Based on our DFT results, we will discuss the enhanced sensitivity of WO_3_ sensors for NO detection by employing the noble metal catalysts.

## Methods

Our DFT calculations were performed using Vienna *ab initio* simulation package (VASP) with the projector augmented wave method [29-32]. For the exchange-correlation energy, we employed the generalized gradient approximation functional of Perdew-Burke-Ernzerhof [33]. The electronic wave functions were expanded in a plane wave basis with an energy cutoff of 400 eV. The WO_3_(001) surface was modeled by a periodic four-atomic-layer slab composing two alternate WO_2_ plus O layers with approximately 16 Å of vacuum in between the slabs. The *k*-space integration was carried out using a Monhkorst-Pack grid [34] of 4 × 4 × 1 *k* points in the surface Brillouin zone of the monoclinic (1 × 1) unit cell whose size is as large as the cubic (2 × 2) unit cell. We relaxed all atoms except the bottom layer along the calculated forces until all the residual force components were less than 0.01 eV/Å. For the interaction of the NO molecule with the clean and metal-deposited WO_3_(001) surfaces, we initially placed the NO molecule about 3.5 Å away from the surfaces and obtained the adsorption structure by fully structural optimization.

## Results and discussion

### NO adsorption on a clean WO_3_ (001) surface

**Figure 2:**
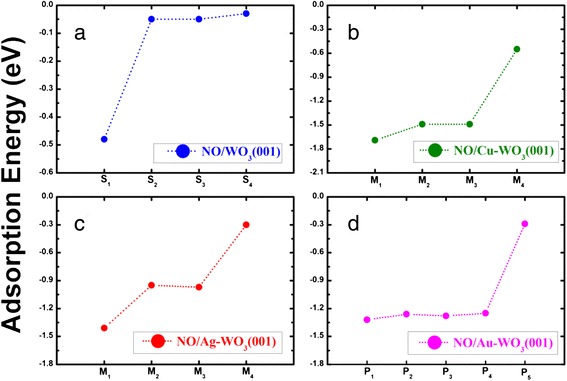
**Adsorption energies of various configurations calculated for NO adsorption.** NO molecule adsorbed on the **(a)** clean, **(b)** Cu-deposited, **(c)** Ag-deposited, and **(d)** Au-deposited WO3 (001) surfaces. The adsorption configurations such as [S_1_, S_2_, S_3_, S_4_], [M_1_, M_2_, M_3_, M_4_], and [P_1_, P_2_, P_3_, P_4_, P_5_] are described in the text.

We first investigate the adsorption of a single NO molecule on a clean WO_3_ (001) surface. Figure 1a,b shows the top and side views of the optimized WO_3_ (001) surface, respectively. For the adsorption of NO on WO_3_ (001), we consider the three different adsorption sites such as top W (hereafter denoted as S_1_), top O (S_2_), and hollow (S_3_) sites. We find that the N atom of NO is bonding to the substrate atoms, consistent with a previous theoretical calculation [21]. However, in the hollow site, the O atom of NO can be bound to the substrate atoms (denoted as S_4_). We calculate the adsorption energy defined as [35] *E*_ads_ = *E*(NO/surf) – *E*(surf) – *E*(NO), where *E*(NO/surf) is the total energy of the NO-adsorbed WO_3_ (001) system, *E*(surf) is the energy of a clean WO_3_ (001) before NO adsorption, and *E*(NO) is the energy of a free NO molecule, obtained using a 12 × 12 × 12 Å^3^ supercell calculation. As shown in Figure 1f, the S_1_ configuration is found to be the most stable with *E*_ads_ = −0.48 eV, larger in magnitude than *E*_ads_ = −0.05, −0.05, and −0.03 eV for S_2_, S_3_, and S_4_, respectively; see Figure 2a. We note that, in the S_1_ configuration, the bond length *d*_N-W_ between the N and W atoms is calculated to be 2.07 Å, which is much shorter than the sum (3.7 Å) of van der Waals radius of the two atoms [14,36]. Thus, we can say that NO molecule can form a chemical bond with the WO_3_ (001) surface.

**Figure 3:**
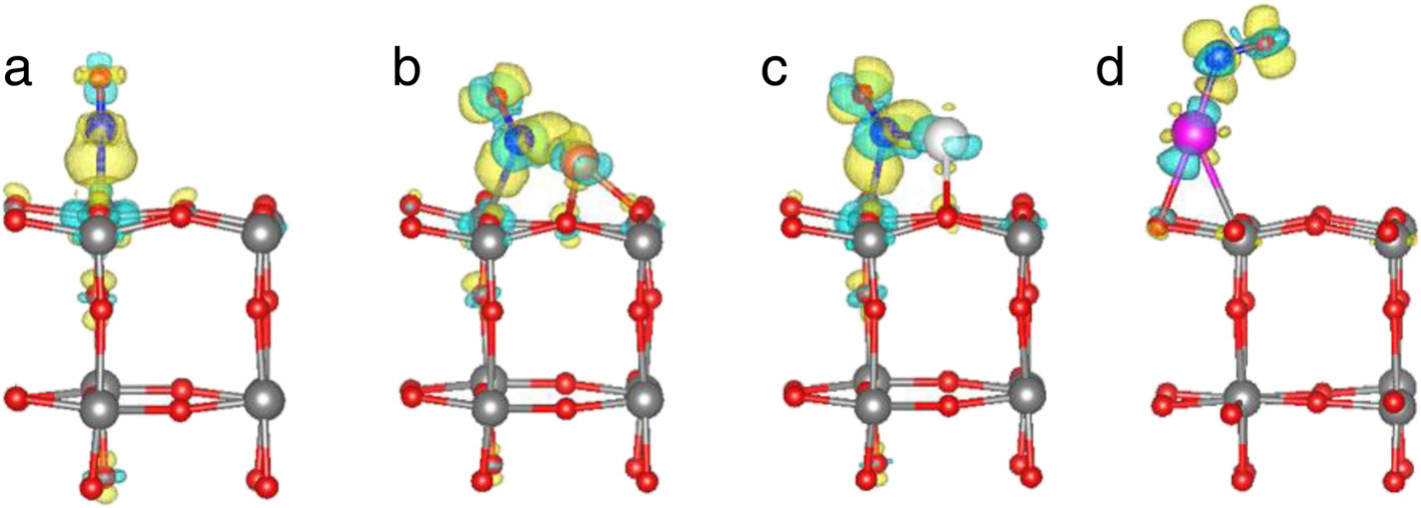
**Charge density difference for the most stable configurations of NO adsorption.** NO molecule adsorbed on the **(a)** clean, **(b)** Cu-deposited, **(c)** Ag-deposited, and **(d)** Au-deposited WO_3_ (001) surfaces. The gain and loss of electrons are drawn in bright and dark colors with an isosurface of 0.005 e/Å^3^, respectively.

To evaluate charge transfer in the S_1_ configuration, we perform Bader charge analysis for NO before and after its adsorption on the WO_3_(001) surface [37,38]. The results for a free NO molecule and adsorbed NO on various substrates are given in Table 1. We find that, upon NO adsorption on a clean WO_3_(001) surface, the electrons in the N (O) atom increase (decrease) from 4.44 (6.56) to 4.84 (6.35) *e*, giving rise to an increase of 0.19 *e* in adsorbed NO molecule. This fact shows that adsorbed NO molecule captures electrons from the WO_3_(001) surface, indicating that NO behaves as a charge accepter. Indeed, the charge density difference, defined as ∆*ρ* = *ρ*_NO/WO3_ − (*ρ*_NO_ + *ρ*_WO3_), clearly shows a charge transfer from the O (in NO molecule) and W atoms to the N atom; see Figure 3a. As a consequence of the additional electrons in NO in the NO/WO_3_(001) system, the bond length *d*_N-O_ of NO molecule slightly increases to 1.181 Å, compared to that (1.170 Å) of a free NO molecule; see Table 1.

**Table 1:** **Charge analysis and bond length of NO molecule**

	**NO**	**NO/WO**_**3**_	**NO/Cu-WO**_**3**_	**NO/Ag-WO**_**3**_	**NO/Au-WO**_**3**_
N (e)	4.44	4.84	5.00	4.91	4.64
O (e)	6.56	6.35	6.43	6.42	6.40
N + O (e)	11	11.19	11.43	11.33	11.04
*d*_N-O_ (Å)	1.170	1.181	1.212	1.203	1.182

Bader charges of N and O atoms in a clean WO_3_(001) surface and various noble metal atom-deposited WO_3_(001) surfaces are given. Bader charges of N and O atoms in an isolated NO molecule are also given in the first column. The bond length *d*_N-O_ in each system is also given.

It is noteworthy that the abovementioned charge transfer from the WO_3_(001) surface to NO molecule leads to a reduction of conduction electrons in WO_3_(001), thereby forming the electron-depleted layer at the surface. This change of electrical character at the WO_3_(001) surface can be utilized to the WO_3_ gas sensor where the contact resistance can be affected by the exposure of NO gas.

### NO adsorption on Cu- or Ag-deposited WO_3_ (001) surface

We begin to optimize the adsorption structure of Cu or Ag on WO_3_(001). We find that the adsorption of Cu (Ag) on the hollow site is more stable than the other adsorption sites such as top W and top O sites by 0.66 (0.14) and 0.75 (0.14) eV, respectively. Using Bader charge analysis, we find that the adsorption of Cu and Ag on the hollow site loses electrons to the WO_3_(001) substrate by 0.7 and 0.6 *e*, respectively. Using the most stable adsorption configuration of Cu or Ag on WO_3_(001), we continue to study the adsorption of NO on such noble metal atom-deposited WO_3_(001) substrates. We consider three different adsorption configurations of NO, where N atom is attached to W (denoted as M_1_), O (M_2_), and Cu or Ag (M_3_) atoms. In addition, we also consider another adsorption configuration of NO, where O atom in NO molecule is attached to Cu or Ag atom (denoted as M_4_). The calculated adsorption energy of NO for each adsorption configuration on Cu-WO_3_(001) and Ag-WO_3_(001) is given in Figure 2b,c, respectively. We find that the M_1_ configuration is the most stable with *E*_ads_ = −1.69 and −1.41 eV for NO/Cu-WO_3_(001) and NO/Ag-WO_3_(001), respectively, which are much larger in magnitude than *E*_ads_ = −0.48 eV of the S_1_ configuration at a clean WO_3_(001) surface. This indicates that Cu and Ag increases the strength of NO binding on WO_3_(001), thereby serving as catalysts. In the M_1_ configuration, the N atom is also bonding to the W atom with *d*_N-Cu/Ag_ (bond length between N and Cu or Ag atoms) = 1.86 or 2.19 Å because of a Coulomb interaction between the negatively charged N atom and the positively charged Cu or Ag atom (see Figure 3b,c), as discussed below. We note that the values of *d*_N-W_ amount to 2.36 and 2.37 Å for NO/Cu-WO_3_(001) and NO/Ag-WO_3_(001), respectively. These values become longer than *d*_N-W_ = 2.07 Å in the S_1_ configuration but are still much shorter than the sum (3.7 Å) of van der Waals radius of N and W atoms [14,36], therefore concluding that NO molecule adsorbs chemically on the Cu-WO_3_(001) and Ag-WO_3_(001) substrates.

In Table 1, we find that for the M_1_ configuration of NO/Cu-WO_3_(001), the electrons in the N (O) atom increase (decrease) from 4.44 (6.56) to 5.00 (6.43) *e*, giving rise to an increase of 0.43 *e* in adsorbed NO molecule. On the other hand, for the M_1_ configuration of NO/Ag-WO_3_(001), the electrons in the N (O) atom are found to increase (decrease) from 4.44 (6.56) to 4.91 (6.42) *e*, giving rise to an increase of 0.33 *e* in adsorbed NO molecule. These results indicate that adsorbed NO molecule on Cu-WO_3_(001) and Ag-WO_3_(001) captures more electrons from the substrates compared to the case of NO adsorption at a clean WO_3_(001) surface, where only 0.19 *e* is transferred from WO_3_(001) to NO. As shown in Figure 3b,c, the calculated charge density difference ∆*ρ* shows charge transfer from the O (in NO molecule) and Cu-WO3(001) or Ag-WO3(001) substrate to the N atom, leading to the polar NO molecule with a negatively charged N atom. We note that, as a consequence of the presence of excess electrons in the polar NO molecule, the bond length *d*_N-O_ of NO molecule increases to 1.212 and 1.203 Å for NO/Cu-WO_3_(001) and NO/Ag-WO_3_(001), respectively. These values of *d*_N-O_ are longer than *d*_N-O_ = 1.181 Å for NO/WO_3_(001) as well as *d*_N-O_ = 1.170 Å of a free NO molecule.

Since more electrons transfer from the substrate to adsorbed NO molecule by the deposition of Cu or Ag atoms, one expects an enhanced reduction of conduction electrons in WO_3_(001), therefore increasing the sensitivity of WO_3_ sensor for NO detection. As a matter of fact, a recent experimental study showed that the deposition of Ag atoms in WO_3_ sensor improves its sensitivity for NO detection [27]. We note that, even though NO adsorption induces more electron transfer from the Cu-WO_3_(001) substrate compared to Ag-WO_3_(001), Cu atoms would be easily oxidized at a usual operation temperature (above 150°C) of WO_3_ sensor. This oxidizing effect in noble metal atoms should be cautioned for the gas-sensing performance of WO_3_ sensor.

### NO adsorption on Au-deposited WO_3_ (001) surface

We first optimize the adsorption structure of Au on WO_3_(001). Unlike the cases of Cu and Au catalysts, Au atom adsorbs only on top of the W atom, as shown in Figure 1e. Here, the adsorption of Au captures electrons from the WO_3_(001) substrate by 0.34 *e* because of a high electronegativity of Au atom. For the adsorption of NO on Au-WO_3_(001), we consider several adsorption configurations of NO, where N atom is attached to Au (denoted as P_1_), top W (P_2_), top O (P_3_), and hollow (P_4_) sites. In addition, we also consider another adsorption configuration of NO, where O atom in NO molecule is attached to Au atom (P_5_). The calculated adsorption energy of NO for each adsorption configuration on Au-WO_3_(001) is displayed in Figure 2d. We find that the P_1_ configuration is the most stable with *E*_ads_ = −1.32 eV, which is relatively smaller in magnitude than *E*_ads_ = −1.69 and −1.41 eV for the M_1_ configurations of NO/Cu-WO_3_(001) and NO/Ag-WO_3_(001), respectively. In the P_1_ configuration, the calculated bond length of adsorbed NO is *d*_N-O_ = 1.182 Å (see Table 1), which is shorter than 1.212 and 1.203 Å for NO/Cu-WO_3_(001) and NO/Ag-WO_3_(001), respectively. This shortest value of *d*_N-O_ is due to the fact that NO captures the least electrons (0.04 *e*) from Au-WO_3_(001), as shown in Table 1. These features of NO/Au-WO_3_(001) such as the smaller adsorption energy, the shorter bond length, and the less electron capture of adsorbed NO is traced to a large electronegativity of Au.

## Conclusions

We have performed first-principles DFT calculations within the generalized gradient approximation for the adsorption of NO molecule on a clean WO_3_(001) surface as well as on the Cu-deposited, Ag-deposited, and Au-deposited WO_3_(001) surfaces. We found that the NO molecule prefers to adsorb on the top of W atom at a clean WO_3_(001) surface, where a charge transfer from WO_3_(001) to NO occurs by 0.19 *e* and *E*_ads_ is calculated to be −0.48 eV. We also found that, on the Cu- and Ag-deposited WO_3_(001) surface, the NO molecule also binds to the W atom with *E*_ads_ = −1.69 and −1.41 eV, respectively, accompanying the relatively larger charge transfer of 0.43 *e* (Cu) and 0.33 *e* (Ag) to adsorbed NO compared to the clean WO_3_(001) surface. On the other hand, Au atoms on WO_3_(001) prefer to adsorb on the top of W atom, and the NO molecule forms a bond to the Au atom with a small electron transfer of 0.04 *e* to adsorbed NO. We obtained a relatively smaller adsorption energy of *E*_ads_ = −1.32 eV for the NO/Au-WO_3_(001) system compared to NO/Cu-WO_3_(001) and NO/Ag-WO_3_(001) because of a large electronegativity of Au atom. The present results demonstrated that the sensitivity of WO_3_ sensors for NO detection can be improved by employing the noble metal catalysts such as Cu and Ag atoms.
